# Effects of Serum Vitamin D Levels and Vitamin D Supplementation on Urticaria: A Systematic Review and Meta-Analysis

**DOI:** 10.3390/ijerph18094911

**Published:** 2021-05-05

**Authors:** Yajia Li, Ziqin Cao, Jia Guo, Qiangxiang Li, Juan Su

**Affiliations:** 1 Department of Dermatology, Xiangya Hospital, Central South University, Changsha 410008, China; zndxlyj1996@csu.edu.cn (Y.L.); jiaguo28@csu.edu.cn (J.G.); 2 Department of Spine Surgery, Xiangya Hospital, Central South University, Changsha 410008, China; xyeyyziqincao@csu.edu.cn; 3 National Clinical Research Center for Geriatric Disorders of Xiangya Hospital, Central South University (Sub-center of Ningxia), Yinchuan 750001, China; liqiangxiang@nxmu.edu.cn; 4 Ningxia Geriatric Disease Clinical Research Center, People’s Hospital of Ningxia Hui Autonomous Region, Yinchuan 750001, China; 5 Hunan People’s Hospital, Department of Hunan Institute of Geriatrics, Changsha 410002, China

**Keywords:** urticaria, vitamin D, supplementation, systematic review, meta-analysis

## Abstract

(1) Backgrounds: Several studies have shown that the level of 25-hydroxyvitamin D (25(OH)D) could affect urticaria. The association of Vitamin D (VitD) with urticaria has not been well established. (2) Methods: The up-to-date meta-analysis was performed to synthesize the new findings. We performed a systematic search in PubMed, EMBASE, Web of Science, and Cochrane Database. We included the observational studies with the comparisons of 25(OH)D between urticarial populations and controls and clinical studies with the clinical severity of urticaria records. (3) Results: A meta-analysis of seventeen studies of urticaria group vs. controls revealed a mean difference of −9.35 ng/mL (95% CI −12.27 to −6.44). There was also an association of urticaria with VitD deficiency. In the subgroup analysis of age and disease type, significant effects of 25(OH)D were found among adult and chronic urticarial populations. Six VitD supplementation trials showed a significant reduction in clinical urticarial score on intervention with VitD with the standard mean difference of −3.63 and −1.54 among randomized control studies and repeated measure trials, respectively. (4) Conclusions: The urticarial population, especially the adult chronic urticarial patients, may be associated with a high risk for lower serum 25(OH)D. VitD supplementations could result in a reduction of urticarial clinical symptoms.

## 1. Introduction

Urticaria is a mast cell-driven skin disorder, clinically characterized by itchy wheals (measles) and/or angioedema. It is a common disease and makes a significant impact on the quality of life. Based on its duration, it is either classified as acute (≤6 weeks) or chronic urticaria (CU) [1]. Urticaria affects the global population, albeit with considerable regional differences in prevalence. Women also tend to be more affected by urticaria than men, and the disease seems more common among adults than among children [2,3,4]. The average prevalence for lifetime acute urticaria is approximately 20%, with a point prevalence range of 0.1–0.6%, whereas for CU, the lifetime risk is 1.4% with a point prevalence rate of 0.7% [1]. Notably, the incidence of urticaria is growing worldwide, and therefore a better understanding of its pathogenesis is urgently needed.

Urticarial etiology appears to be highly heterogeneous, involving a complex interplay between mast cell-activating signals, Th1/Th2 macrophage imbalance, and defects in regulatory T function, all likely contributing to the systemic inflammation. Multiple factors, including physical irritation, air allergens, food and food additives, contact allergens, drugs, and infectious pathogens, have been suggested to contribute to urticaria [5,6,7]. However, the key factors affecting the long-term remission of urticaria are currently unclear. Presently, quantitative and qualitative clinical tools are used to measure the severity of clinical manifestations. The measurements, including the urticaria activity score (UAS), urticaria severity score (USS), the angioedema quality of life questionnaire (AE-QoL), the CU quality of life questionnaire (CU-Q2oL) in both observational and clinical studies, have contributed to better defining the effects of urticaria [8].

Previous studies have demonstrated that Vitamin D 3 (VitD 3) has an important role in skin diseases; for example, it can modulate structural proteins in the cornified dermis layer and affects skin barrier disorder-related diseases such as atopic dermatitis. VitD enhances the number and immunosuppressive activity of regulatory T (Treg) cells while inhabiting normal T cell function [9]. It also acts as a protective factor in allergy by inhibiting the production of pro-inflammatory cytokines such as interleukin (IL)-1, IL-6, IL-12, and IFN-γ. Alternatively, VitD enhances tolerogenic cytokine production such as IL-10 and TGF-β by Tregs, dendritic cells (DCs), also affecting the proliferation, survival, differentiation, and function of mast cells [10,11,12,13].

Supported by previous research, there is much interest in the role of VitD deficiency in the development of urticaria. Serum VitD (25-hydroxyvitamin D or 25(OH)D levels tend to be lower in the winter, and there is a significant seasonal pattern (winter to spring) of acute urticaria (AU) with temperature inversely associated with incidence [14,15]. Moreover, improvements in urticarial symptoms have been reported after VitD supplementation [16,17]. Interestingly, polymorphisms in the vitamin D receptor (VDR) gene such as the SNPs rs1544410 and rs2228570 alter VDR function and have been frequently studied in association with allergic diseases [18]. Furthermore, Nasiri–Kalmarzi et al. concluded that gene or protein alterations affecting the VitD pathway might alter the risk of CU. Thus, more studies are warranted to evaluate the relationship between 25(OH)D status and urticarial patients [19].

Bone health recommendations of the US Endocrine Society divide serum 25(OH)D levels into three categories [20]: deficiency (<20 ng/mL), insufficiency (20–30 ng/mL), and sufficiency (>30 ng/mL). However, the optimum 25(OH)D levels to prevent skin allergies such as urticaria or other skin diseases remain to be determined. Two previous systematic reviews and meta-analyses [21,22] revealed a lower serum 25(OH)D level was found in urticarial patients compared with controls. Significant new studies have been conducted since these system reviews. Presently, there is a clear demand for an updated systematic review and meta-analysis. 

Our study aimed to review the latest observational and intervention study data on the role of VitD in urticarial and to systematically synthesize the magnitude of associations, including studies published up to March 2021. We assess observational studies comparing serum VitD levels in urticarial patients and healthy controls, as well as the association of VitD inadequacy with urticaria. Furthermore, we report on new research linking the impact of VitD supplementation on urticarial severity and patient life quality, also assessing the supporting evidence of clinical treatments. The meta-analysis protocol was conducted according to the Preferred Reporting Items for Systemic Reviews and Meta-Analysis (PRISMA) guidelines. 

## 2. Materials and Methods

### 2.1. Data Sources and Search Strategy

We performed a systematic search on the database via PubMed, EMBASE, and Cochrane Database from January 1990 to January 2021. We also searched for literature such as authoritative information from letters to editors and abstracts from conference presentations. For search strategies, Medical Subject Headings (MESH) and pre-text terms were used, and search terms included: “Vitamin D AND Urticaria”; “Vitamin D AND nettle-rash”; “25 Hydroxy Vit D AND Urticaria”; “Vitamin D AND Urticaria AND children”. The search was limited to English-language studies with no restriction on the year of publication. We Identified other related articles by consulting the reference list and searching for related papers. The search procedure is documented using the PRISMA protocol, which was registered in PROSPERO (CRD42021236551).

### 2.2. Study Selection

Two investigators (Y.L. and Z.C.) independently assessed article titles and abstracts to determine eligibility for inclusion in the meta-analysis. Disagreements were addressed through discussion with the third investigator (J.G.). The inclusion criteria for original studies involved: studies had to be observational or interventional, include randomized control trials (RCT), non-randomized control trials, clinical intervention studies, prospective case-control studies, and repeated measure studies. We excluded studies where participants were pregnant women or infants (<1 year); involved case series, case reports, literature reviews, or commentaries. Furthermore, studies had to compare two groups (case group composed of urticarial patients and control group composed of healthy individuals without urticaria); the diagnosis of urticaria and the measurement of serum 25(OH)D had to be documented; participants with different serum VitD levels (sufficiency/insufficiency/deficiency) could be assessed and numerically reported (numbers of affected patients in case/control group, percent values, and Odds Ratio (OR)). Intervention studies were only included, involving assessment of baseline severity of urticaria and serum 25(OH)D levels, which help provide calculable data for evidence of improvement or exacerbation. 

### 2.3. Data Extraction and Quality Assessment

Both authors confirmed all entries and verified the accuracy and completeness of data. The following information was extracted: authors, publication year, geographic location by latitude, study design, urticarial type, participants type, patient demographic characteristics. For observation studies, we also extracted quantitative estimates, including the mean ± standard deviation (SD) of 25(OH)D levels (ng/mL) of case/control groups. Serum 25(OH)D concentrations in two studies presented in nmol/L were converted to ng/mL using the standard formula: 2.5 nmol/L = 1 ng/mL. The number of the cases of VitD status of below sufficiency (<30 ng/mL) were also from both groups. For intervention studies, data (mean ± SD) of serum 25(OH)D levels, urticaria severity assessment, life quality scores were obtained both in baseline and after the intervention, as well as the dose of VitD and study duration.

We used the Newcastle-Ottawa Quality Assessment Scale (NOS) [23] and Jadad Quality Scale [24] to measure the quality of observation and interventional studies. In the NOS system, we used score categories of 0 to 3 (low quality), 4 to 6 (moderate quality), and 7 to 9 (high quality), respectively. The Jadad tool assessed the risk of bias from six domains: sequence generation, allocation concealment, blinding, incomplete outcome data, selective outcome reporting, and other sources of bias. Results are presented with a low, unclear, or high risk of bias. The Evidence-based medicine (EBM) guidelines are summarized in a level of evidence (LOE) table and consist of 5 levels (I–V) [25]. We ranked the evidence level for each study included in the meta-analysis.

#### 2.3.1. Primary Outcomes

1. For observational studies, serum 25(OH)D levels in urticarial patients versus healthy controls; the prevalence of below the VitD sufficiency level (<30 ng/mL) urticarial patients versus HC;
2. For interventional studies, changes in urticaria severity in the VitD supplemented urticarial group compared to the control group or baseline.

#### 2.3.2. Secondary Outcomes

1. Changes in post-supplemented serum 25(OH)D levels in the VitD supplemented urticarial group compared to baseline;
2. From interventional trials, urticarial-related life quality change in the VitD supplemented urticarial group compared to the control group or baseline levels.

### 2.4. Data Analysis

For observational studies, the mean ± standard deviation (SD) of serum 25(OH)D levels of urticarial and healthy control groups were extracted as ng/mL and imputed into the meta-analysis. We also calculated the unadjusted ORs for VitD below sufficiency (<30 ng/mL), which could be divided into insufficiency (20–30 ng/mL) group and deficiency (<20 ng/mL) group, for case-control studies with pooled OR reported with 95% confidence intervals (CIs). Mean difference (MD) was used when the assessment standard and unit of measurements were consistent. Standard mean difference (SMD) was calculated to calculate the change in outcomes measured by different tools and units.

Heterogeneity between studies was assessed by the I^2^ statistic. Because of significant heterogeneity found in between-study, the random-effects model was used in meta-analysis.

The meta-analysis of interventional studies used changes in the urticaria severity score before and after VitD supplementation, and the weighted average dose was manually calculated in the meta-analysis by multiplication of each trial dose through given weight in the meta-analysis and summing-up of the dosage to represent the 100% weighted mean dose. A series of sub-analyses were also undertaken using data from the two studies with data for separate age groups (adult or child), different urticarial types (chronic or acute), and study locations by latitude (low/middle/high). To assess potential publication bias, we employed funnel plots and also used the Begg and Egger tests. We also performed a sensitivity analysis by excluding studies one by one to explore sources of study heterogeneity. All analyses were performed using the Review Manager version 5.3 (The Nordic Cochrane Centre, The Cochrane Collaboration, London, UK) and STATA (version 14.0; StataCorp LLC, College Station, TX, USA).

## 3. Results

### 3.1. Study Characteristics

The PRISMA diagram presented in Figure S1 shows the inclusion of 22 articles published between 2011 and 2020, including 17 observational studies (two prospective case-control studies with repeated measure trials data) and five interventional studies (one study with case-control data). Among the total of 7790 participants from observational studies, there were 2007 urticarial patients and 6498 healthy controls. From interventional trials, 242 participants have received VitD treatments. Study settings included large outpatient databases and inpatient cohorts. Patient characteristics from the observational and interventional studies are shown in Table 1 and Table 2, respectively.

### 3.2. Association of Serum Vitamin D Levels with Urticaria

#### 3.2.1. Comparison of Serum 25(OH)D Level

**Figure 1:**
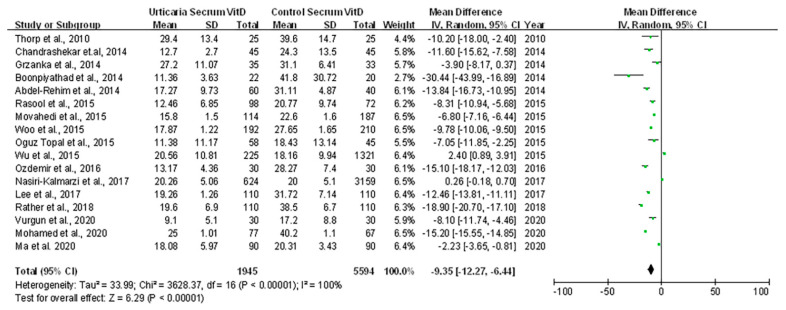
Forest plot for meta−analysis of serum 25(OH)D levels in the urticarial population (ng/mL).

A total of 7539 eligible participants were used to compare serum 25(OH)D in urticarial patients against healthy controls (Table 3). This analysis revealed a statistically significantly lower level of 25(OH)D in urticarial patients compared with controls (−9.35 ng/mL, 95% CI −12.27 to −6.44, *p* = 0.001; I ^2^ = 100%). Very high heterogeneity was detected, so a random effect model was used. (Figure 1)

In the subgroup-analysis (Figure S2), two studies involved a mixed adult population and pediatric populations, another two studies involved only pediatric subjects, whereas the other 13 studies documented adult populations. The sub-group results were mostly in accordance with the meta-analysis where urticarial patients from mixed or adult study populations had lower levels of serum 25(OH)D by −8.02 ng/mL and −9.78 ng/mL, respectively. However, no differences were seen in the pediatric-only group (−7.34 ng/mL, 95% CI −22.39 to 7.71, *p* = 0.34). (Figure S2a) Assessment of different urticarial types indicated that chronic urticarial patients displayed significant reductions of −8.72 ng/mL in 25(OH)D levels. However, this trend was not evident in AU patients with a mean difference of −7.16 (95% CI −16.53 to 2.20, *p* = 0.13). (Figure S2b) Those effect sizes for the subgroups of children and AU should be explained with caution because, at present, only a few studies with a small population could be included studies.

The skin can make little vitamin D from the sun at latitudes above 37 degrees north or below 37 degrees south of the equator except during the summer months [45,46]. Using the N37° as a cut-off value, we divided study participants into high and low latitude groups. In subgroup divided by latitude, seven studies conducted in low latitude area showed lower levels of serum 25(OH)D of −10.48 (95% CI −14.23 to −6.73, *p* = 0.001) in urticarial population than controls, while in high latitude areas, the mean difference was −7.72 (95% CI −14.09 to −1.35, *p* = 0.001) (Figure S2c).

#### 3.2.2. VitD Deficiency and Urticaria

**Figure 2:**
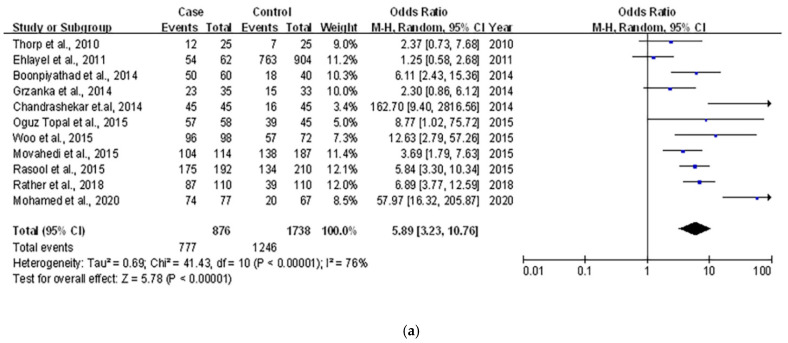

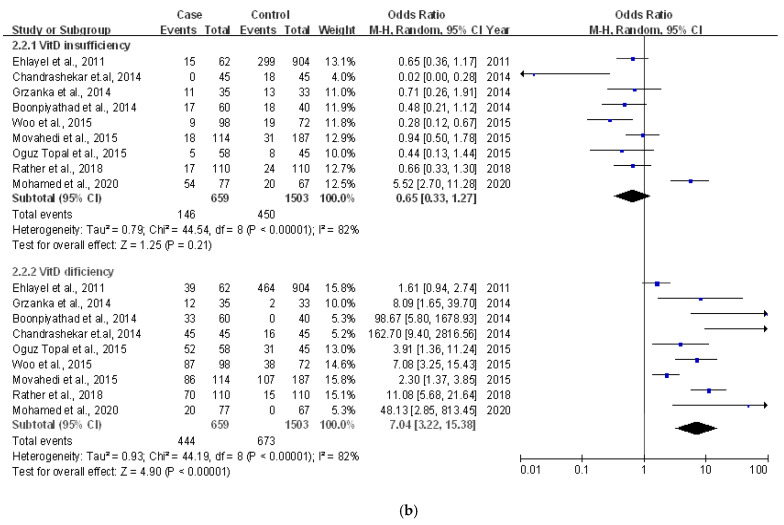
(**a**) Forest plot for the association of serum 25(OH)D below the level of sufficient (30 ng/mL) with urticaria. (**b**) Forest plot for the association of serum 25(OH)D insufficiency or deficiency with urticarial.

We also compared the cases of below sufficient VitD levels (<30 ng/mL) in urticarial and control groups from 2614 participants included in 11 studies (Table 3). The pooled OR for the association between urticaria and prevalence of VitD insufficiency was 5.89 (95% CI 3.23 to 10.76, *p* < 0.001; I ^2^ = 77%). (Figure 2a) Of note, we also specifically divided the VitD level into insufficiency (20–30 ng/mL) and deficiency (<20 ng/mL) based of the extent, and their respective pooled ORs for the association between urticarial were 0.65 (95% CI = 0.33 to 1.27; *p* = 0.21) and 7.04 (95% CI = 3.22 to 15.38; *p* = 0.001). Only two studies reported the total number of below sufficient VitD levels without the respective amount for two groups. (Figure 2b).

As the main results showed serum 25(OH)D deficiency was associated with urticaria, the analysis was subgrouped by urticarial type, and the result showed that VitD deficiency was significantly associated with CU (OR = 11.48, 95% CI 4.26 to 30.95, *p* < 0.001) but similar differences were not observed in patients with acute urticaria (OR = 2.94, 95% CI = 0.72 to 12.05, *p* = 0.13). (Figure S3a)Studies were also distinguished by the latitude. Compared with high latitude area with the pooled OR of 4.88 (95% CI = 2.03 to 11.77, *p* < 0.001), the subgroup analysis showed that VitD was also associated with urticaria (OR = 8.13, 95% CI = 3.11 to 21.29, *p* < 0.001) in low latitude areas (Figure S3b).

We conducted a meta-analysis for six interventional studies (including three RCTs and three repeated measure trials) in chronic urticarial cases with the primary outcome of change in clinical urticarial severity after VitD supplementation compared to the baseline value. Three studies assessed clinical severity by the Urticaria Severity Score (USS), two used the Urticaria activity score (UAS)-7 [30,43], and one [33] used the UAS-4 measurement. Two subgroups were used to perform the analysis because the studies of repeated measures used self-controls (where patients are their control) and could not be statistically combined with RCTs. Outcomes were shown in standard mean difference (SMD) because different assessment tools of urticarial clinical severity were used (Table 4).

#### 3.2.3. Interventional Vitamin D Supplements and Changes in Clinical Urticarial Assessments

**Figure 3:**
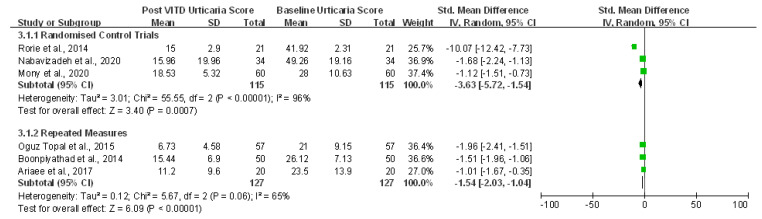
Forest plot for vitamin D intervention trials in urticaria: Comparison of clinical severity score at baseline and post-vitamin D supplementation compared with healthy controls (ng/mL).

For RCTs, there was a highly robust reduction in clinical urticarial scores upon VitD intervention (SMD = −3.63, 95% CI −5.72 to −1.54, *p* < 0.001; I 2 = 96%) with an estimated weighted mean dose of 4106.85 IU/day. Similarly, for the repeated measures interventions, VitD treatment was associated with significant reductions in clinical urticarial scores (SMD = −1.54, 95% CI −2.03 to −1.04, *p* < 0.001; I 2 = 65%) with an estimated weighted mean dose of 12888.34 IU/day (Figure 3). The random-effect model analysis revealed potential heterogeneity among studies; however, while there were a limited number of studies, all showed improvement in clinical urticarial severity indices with VitD supplementation. Moreover, the serum 25(OH)VitD levels among urticarial patients were also significantly improved after supplementation with VitD in the interventional studies (Figure S4a). Only three studies evaluated life quality, two using the Dermatology Life Quality Index (DLQI) [30,33], and one [44] assessing the chronic urticaria quality of life questionnaire (CU-Q2 oL). A statistically significant reduction was observed in life quality scores after VitD supplementation in the meta-analysis (Figure S4b).

### 3.3. Risk of Bias

The observational studies included in our meta-analysis were rated as low risk of bias according to the NOS (Table S1a), and Quality analysis of the interventional studies using the Jadad scale is shown in Table S1b, with most studies were ranked as low risks. Funnel plots of the analyses revealed possible publication bias because the positive effect sizes were shown in relatively fewer research. For example, there were few results that serum 25(OH)D higher in the case group compared with healthy control. (Figure S5a,b). However, results of the Begg (*p* = 0.207) and the Egger tests (*p* = 0.085) indicated no significant publication bias in studies evaluating the association of serum VitD level with urticaria. Likewise, no significant publication bias was detected for studies relating to VitD deficiency and urticaria, according to the Begg (*p* = 0.586) and the Egger tests (*p* = 0.339).

### 3.4. Sensitivity Analysis

Sensitivity analyses were performed by assessing the relative impact of each study by the exclusion approach, indicating all of the results used in the meta-analysis were stable (Supplemental Figure S5c,d) where each study only had a small influence on the effect size with no effect on statistical significance.

## 4. Discussion

The findings of our comprehensive meta-analysis showed lower serum 25(OH)D concentrations by 9.35 ng/mL in the overall urticarial population compared with healthy individuals. Moreover, serum 25(OH)D deficiency was associated with an increased prevalence of urticaria compared to the general population. Many previous studies pointed out the positive relationship between low VitD serum levels and urticaria. Compared to the most recent meta-analyses in this field, our study concurs with the results by Tsai et al. (2018) [22] and Wang (2018) [21] that lower VitD serum levels occur in urticarial patients. We also verified that VitD deficiency rather than insufficiency has a positive relationship with urticaria. Furthermore, the higher prevalence of VitD deficiency and lower serum VitD levels were only found in patients with CU but not in those with AU. There lacked association of serum Vit D levels with AU and children urticaria compared to CU and adults (Supplemental Figure S6). Neither can we ignore the impact of geographical location, where subgroup analysis indicated populations in lower latitudes with VitD deficiency were associated with a higher urticarial risk compared to those in high latitudes. According to previous studies, sun exposure is an important source of the human needs for cutaneous production of VitD. Ultraviolet B (UVB) could be absorbed and transformed to pre-vitamin D3 in the skin by the function of 7-dehydrocholesterol, which could be influenced by many factors such as season, latitude, time of day, etc. [47]. For the effects of latitude or sun exposure on serum VitD level, notably, people who were below 37° and closer to the equator could synthesize more VitD3 in their skin within a year. In the early morning or evening, the apex angle of the day was so inclined that there was almost no VitD3 produced in the skin, even in summer. Therefore, studies have shown that safe exposure to the sun between 1000 and 1500 h in spring, summer, and autumn is considered to be critical for VitD generation because this is the only time that sufficient solar UVB reaches the earth’s surface to produce VitD3 in the skin [48,49,50]. However, In addition to latitude, other critical determinants of VitD status may not have been adequately captured in our analysis, including seasonal and personal factors.

As noted above, our meta-analysis was different from prior studies since we also focused on the effects of VitD supplementation on urticaria. Pooled results from the repeated measures study indicated there were highly significant improvements after supplementation with reductions in urticarial severity scores of −1.54, using dosages of 7000–20,000 IU daily for 6–12 weeks. Similarly, pooling results from RCTs showed highly significant improvements after VitD supplementation with changes of severity by −3.63, using dosages around 4000 IU daily for 12 weeks. For all interventional studies used, the baseline average serum 25(OH)D was below 30 ng/mL and therefore below sufficient levels. Thus, we were not able to reveal the effects of supplementation in populations with sufficient serum 25(OH)D status. Nonetheless, we found that after supplementation, the mean 25(OH)D levels were >30 ng/mL in all trials except one with a mean concentration of 29 ng/mL. The weighted mean dose of 4000–12,000 IU/day (100–300 micrograms) was high in comparison with existing treatments, although no complications were reported [51]. Except for one study that did not describe the formulation of VitD supplementation used, only Boonpiyathad et al. [30] reported the use of VitD_2_, which can be metabolized more rapidly, which might explain why VitD_2_ is less effective than VitD_3_ in increasing 25(OH)D concentrations [52]. However, it was assumed that large amounts of VitD_2_ might have fewer toxic effects than VitD_3_ [53]. The optimum dose for effectiveness and safety of VitD supplements may be required in future studies.

In terms of the biological mechanisms involved, Ariaee et al. found that VitD treatment was associated with downregulation of IL-10, TGF-beta, FOXP3, and IL-17 in which type of cells [17]. Although these were not statistically significant, there was evidence revealing that a Th17/Treg cell subsets imbalance was reported to be involved in the pathogenesis of CSU and have a functional role in relieving diseases. Skin inflammation could be relieved by the balance of Th17/Treg cell populations [54,55]. VitD also could inhibit migration of DCs and decrease IL-6, IL-12, IL-23, C-reactive protein, TNF-a, and IgE production [56,57,58,59]. Besides, the active form of VitD could downregulate Th1 gene expression as well as up-regulate Th2 gene expression, leading to a Th2 response and increased production of IL-4, IL-5, and IL-10 [17,60]. All these actions could plausibly contribute to the role of VitD in urticaria. However, few studies have been published, and more research is needed to better define the mechanisms.

Our study currently represents the most up-to-date meta-analysis concerning the role of VitD in urticaria in the broader global population, with most included studies scored as high quality. Our study is also the first for assessing clinical changes in urticarial disease severity and life quality after VitD supplementation. However, even considering publication bias, several limitations must be acknowledged concerning our study. First, the interventional studies used different urticaria severity scores, and some studies used self-control or non-placebo controls. Second, all studies involved high-dose VitD treatments, and we were unable to evaluate dosage effects. Third, the measurement tools for severity and life quality were different amongst interventional studies. Last, among both observational and interventional studies, the meta-analysis, especially for subgroups, was in limitations because there were small populations that were appropriate for inclusion. For example, Only a few studies about AU were included and may cause bias in the results.

Besides, there some evidence showing that substantial seasonal variations could be found in the 25(OH)D concentrations. Hansen et al. showed that among the general Danish population, most people have relatively sufficient VitD in the summer or fall, but they are more prone to VitD deficiency in the winter or early spring. Individualized and bi-seasonal measurements seemed necessary when assessing serum VitD status. The seasonal variations in 25-hydroxyvitamin D concentration varied in different regions in the world and could be affected by complex factors. With the lacked seasonal information on serum collection in most studies, it is difficult to compare VitD level and conditions among urticaria patients and healthy controls subgrouped by different seasons. The problem in all studies of linking vitamin D status to a disease process, that there is considerable seasonal variation in vitamin D status and that the season when blood samples are collected is often not stated. More related studies with descriptions about the season for blood sample collection are warranted.

## 5. Conclusions

In summary, our findings support that the urticaria population, especially the adult chronic urticaria patients, may be at high risk associated with lower serum 25(OH)D. VitD, as an immunomodulatory and anti-inflammatory agent, can benefit chronic urticaria. VitD supplementation appears to both reduce urticaria severity and improve life quality. Regardless, large multi-center, long-duration clinical studies are still required to investigate the clinical benefits and to understand the mechanisms of function of VitD in urticaria.

## Figures and Tables

**Table 1:** Observation studies of serum 25(OH)D levels in urticaria individuals compared to healthy controls.

Study	Study Design	Geographical Location	Latitude	Urticarial Type	Sample Size			Mean Age		Sex (Male: M; Female: F)	Population (Adult:A; Child: C)	Evidence Level *
					N	*n*-Cases	*n*-Controls	Cases (yrs)	Controls (yrs)			
Thorp et al. [26] 2010	case-control	US (Nebraska)	high	CU	50	25	25	42.4 ± 12.8	42.1 ± 12.2	NA	A	III
Ehlayel et al. [27] 2011	case-control	Qatari (Hamad)	low	AU	966	62	904	Total: 7.7 ± 3.77		NA	C	III
Chandrashekar et al. [28] 2014	case-control	South India (Puducherry)	low	CU	90	45	45	30.2 ± 8.2	29.0 ± 7.1	M: 32; F: 58	A	III
Grzanka et al. [29] 2014	case-control	Poland (Zabrze)	high	CSU	68	35	33	35.3 ± 6.89	NA	M: 32; F: 58	A	III
Abdel-Rehim et al. [16] 2014	case-control	Egypt (Cairo)	low	CSU	42	22	20	32.8 ± 13.17	NA	NA	A	III
Boonpiyathad et al. [30] 2014	case-control	Thailand (Bangkok)	low	CSU	100	60	40	37 ± 10	43 ± 17	M: 28; F: 72	A	II
Wu et al. [31] 2015	case-control	UK (Southampton)	high	CSU	1546	225	1321	Total: 41.2 ± 12.41		M: 613; F: 933	A	III
Rasool et al. [32] 2015	case-control	India (Srinagar)	low	CSU	402	192	210	42.83 ± 8.52	45.12 ± 7.65	NA	A	III
Oguz Topal et al. [33] 2015	case-control	Turkey (Istanbul)	high	CU	103	58	45	40.09 ± 14.59	NA	M: 81; F: 22	A, C	II
Woo et al. [34] 2015	case-control	Korea (Daejeon)	low	CU and AU	170	98 (CU: 72; AU: 26)	72	CU: 37.89 ± 16.13 AU: 29.07 ± 17.07	38.61 ± 15.12	M: 68; F: 102	A, C	III
Movahedi et al. [35] 2015	case-control	Iran (Tehran)	low	CSU	301	114	187	29.4 ± 1.3	32.8 ± 1	M: 42; F: 259	A	III
Ozdemir et al. [36] 2016	case-control	Turkey (Ankara)	high	AU	60	30	30	2.78 ± 1.28	2.73 ± 1.23	M: 36; F: 24	C	III
Lee et al. [37] 2017	case-control	Korea (Seongnam)	high	CU and AU	3783	624 (CU: 567; AU: 57)	3159	9.40 ± 1.72	9.4 ± 1.79	M: 1953 F: 1830	C	III
Nasiri-Kalmarzi et al. [19] 2017	case-control	Iran (Sanandaj)	low	CU	220	110	110	34.79 ± 1.06	31.20 ± 0.76	M: 81; F: 109	A	III
Rather et al. [38] 2018	case-control	India (Jammu and Kashmir)	low	CU	220	110	110	41.82 ± 8.51	43.11 ± 7.54	M: 78; F: 142	A	III
Vurgun et al. [39] 2020	case-control	Turkey (Istanbul)	high	CSU	60	30	30	41.5	32	M: 16; F: 44	A	III
Mohamed et al. [40] 2020	case-control	Egypt (Cairo)	low	CSU	144	77	67	36.5 ± 5.12	39.3 ± 7.23	M: 65; F: 79	A	III
Ma et al. [41] 2020	case-control	China (Nanjing)	low	CSU	180	90	90	34.87 ±11.69	35.7 ± 10.18	M: 74; F: 106	A	III

Note: * The Evidence-based medicine (EBM) guidelines are summarized in a level of evidence (LOE) table and consist of five levels (I–V).

**Table 2:** Interventional studies of vitamin D supplementation in urticaria.

Study	Study Design	Geographical Location	Type of Urticaria	Mean age (yrs)	Sex (Male: M; Female: F)	Population (Adult: A; Child: C)	VitD Supplementation		Percentage Weight of Study as Per Forest Plot (%)	Calculation of Weighted Mean Dose per Study (IU/day)	Period	Evidence Level *
							Interventions	Dose (IU/day)				
Rorie et al. [42] 2014	RCT	U.S. (Nebraska)	CU	43.5	M: 9; F: 33	A	vitamin D 3	4000	7.4	296.0	12 weeks	I
Boonpiyathad et al. [30] 2014	Prospective case-control (with Repeated Measures data)	Thailand (Bangkok)	CSU	37.0	M: 28; F: 72	A	Ergocalciferol (vitamin D2)	20,000	18.8	3760.0	6 weeks	II
Oguz Topal et al. [33] 2015	Prospective case-control (with Repeated Measures data)	Turkey (Istanbul)	CU	40.09	M: 13; F: 45	A, C	Vitamin D3	10,000	18.8	1880.0	12 weeks	II
Ariaee et al. [17] 2017	Clinical trial	Iran (Mashhad)	CSU	35.6	M: 9; F: 11	A, C	Oral Vitamin D	Estimated 7142	17.6	1257.0	8 weeks	II
Mony et al. [43] 2020	RCT	India (Pondicherry)	CU	37.76	M: 25; F: 95	A	Vitamin D 3	Estimated 4285.7	19.1	818.6	12 weeks	I
Nabavizadeh et al. [44] 2020	RCT	Iran (Shiraz)	CSU	40.0	M: 11; F: 58	A	Vitamin D 3	4000	18.2	728.0	12 weeks	I
							Weighted Mean Dose		100	8739.6		

Note: * The Evidence-based medicine (EBM) guidelines are summarized in a level of evidence (LOE) table and consist of five levels (I–V).

**Table 3:** Showing data of serum 25(0H)D levels in the urticaria participants compared to healthy controls in included studies.

Study	Serum 25(OH)VitD Levels (ng/mL)-Cases	Serum 25(OH)VitD Levels (ng/mL)-Controls	Cases		Control	
			Events of VITD < 30 ng/mL * (Insufficiency: I; Deficiency: D)	Total	Events of VITD < 30 ng/mL (Insufficiency: I; Deficiency: D)	Total
Thorp et al. 2010	29.40 ± 13.40	39.60 ± 14.7	Total: 12	25	Total: 7	25
Ehlayel et al. 2011	NA	NA	Total: 54 (I: 15; D: 39)	62	Total: 763 (I: 299; D: 464)	904
Rajappa et al. 2014	12.7 ± 2.7	24.3 ± 13.5	Total: 45	45	Total: 16	45
Grzanka et al. 2014	27.2 ± 11.07	31.1 ± 6.41	Total: 23 (I: 11; D: 12)	35	Total: 15 (I: 2; D: 13)	33
Abdel-Rehim et al. 2014	11.36 ± 3.63	41.8 ± 30.72	NA	22	NA	20
Boonpiyathad et al. 2014	17.27 ± 9.73	31.11 ± 4.87	Total: 50 (I: 17; D: 33)	60	Total: 18 (I: 18; D: 0)	40
Wu et al. 2015	20.56 ± 10.81	18.16 ± 9.94	NA	225	NA	1321
Rasool et al. 2015	17.87 ± 1.22	27.65 ± 1.65	Total: 175	192	Total: 134	210
Oguz Topal et al. 2015	11.38 ± 11.17	18.43 ± 13.14	Total: 53 (I: 5; D: 52)	58	Total: 39 (I: 8; D: 31)	45
Woo et al. 2015	12.46 ± 6.85	20.77 ± 9.74	Total: 96 (I: 9; D: 87)	98	Total: 57 (I: 19; D: 38)	72
Movahedi et al. 2015	15.8 ± 1.5	22.6 ± 1.6	Total: 104 (I: 18; D: 86)	114	Total: 138 (I: 31; D: 107)	187
Ozdemir et al. 2016	13.17 ± 4.36	28.27 ± 7.4	NA	30	NA	30
Lee et al. 2017	20.26 ± 5.06	20 ± 5.1	NA	624	NA	3159
Nasiri-Kalmarzi et al. 2017	19.26 ± 1.26	31.72 ± 7.14	NA	110	NA	110
Rather et al. 2018	19.6 ± 6.9	38.5 ± 6.7	Total: 87 (I: 17; D: 70)	110	Total: 39 (I: 24; D: 15)	110
Vurgun et al. 2020	9.1 ± 5.1	17.2 ± 8.8	NA	30	NA	30
Mohamed et al. 2020	25 ± 1.01	40.2 ± 1.1	Total: 74 (I: 54; D: 20)	77	Total: 20 (I: 20; D: 0)	67
Ma et al. 2020	18.08 ± 5.97	20.31 ± 3.43	NA	90	NA	90

Note: * Vitamin D status defined as a 25(OH)D: Deficiency < 20 ng/mL (50 nmol/L); Insufficiency 21–29 ng/mL (52.5–72.5 nmol/L); Sufficiency >30 ng/mL (75 nmol/L); NA: Not applicable.

**Table 4:** Data of interventional studies included in the meta-analysis.

Study	*n*- Cases	*n*- Control	Serum 25(OH)D Levels		Urticaria severity			Quality of life		
			Baseline (ng/mL)	After VitD Intervention (ng/mL)	Measurements	Scores before Intervention	Scores after Intervention	Measurements	Scores before Intervention	Scores after Intervention
Rorie et al. [42] 2014	21	21	28.8 ± 2.2	56 ± 3.9	USS	41.92 ± 2.31	15.0 ± 2.9	NA	NA	NA
Boonpiyathad et al. [30] 2014	50	50	13.96 ± 4.68	40.88 ± 7.58	UAS7	26.12 ± 7.13	15.44 ± 6.90	DLQI	13.79 ± 6.02	6.79 ± 4.23
Oguz Topal et al. [33] 2015	57	57	NA	NA	UAS4	21 ± 9.15	6.73 ± 4.58	DLQI	41.69 ± 27.3	12.5 ± 9.46
Ariaee et al. [17] 2017	20	20	NA	NA	USS	23.5 ± 13.9	11.2 ± 9.6	NA	NA	NA
Mony et al. [43] 2020	60	60	14.24 ± 2.73	29.07 ± 8.81	UAS7	28 ± 10.63	18.53 ± 5.32	NA	NA	NA
Nabavizadeh et al. [44] 2020	35	34	19 ± 10	46 ± 19	USS	49.26 ± 19.16	15.96 ± 19.96	CU-Q2 oL	60.0 ± 21.25	34.2 ± 15.28

**Note:** USS: urticaria severity score; UAS: urticaria activity score; NA: Not applicable.

## Data Availability

The datasets used and/or analyzed during the current study are available from the corresponding author on reasonable request.

## Supplementary Materials

The following are available online at https://www.mdpi.com/article/10.3390/ijerph18094911/s1, Figure S1: PRISMA flow diagram; Figure S2: Forest plot for sub-analysis of serum vitamin D level comparison. (**a**) Subgrouped by age (**b**) Subgrouped by disease type (**c**) Subgrouped by latitude; Figure S3: Forest plot for sub-analysis of vitamin D deficiency and urticaria. OR: odds ratio; (**a**) Subgrouped by disease type (**b**) Subgrouped by latitude; Supplemental Figure S4. Forest plot for changes in clinical urticarial Assessments after vitamin D supplementation. (**a**) changes in serum 25(OH)VitD levels (**b**) changes in life quality scores; Figure S5: Risk of bias. (**a**) Funnel plots of comparison of serum 25(OH)D levels (**b**) Funnel plots of associations between vitamin D below the level of sufficiency and urticaria. (**c**) Sensitivity analysis for comparison of serum 25(OH)D levels. (**d**) Sensitivity analysis for associations between vitamin D below the level of sufficiency and urticarial; Figure S6: The lack of association of serum vitamin D levels with acute urticaria and children urticaira. Table S1. The methodological quality of studies included in the final analysis. (**a**) Quality of the case-control studies based on the Newcastle–Ottawa Scale. (**b**) Quality of the clinical trials based on the Jadad scale.
