# Supplementary material for: Effects of Serum Vitamin D Levels and Vitamin D Supplementation on Urticaria: A Systematic Review and Meta-Analysis
Source: Int J Environ Res Public Health. 2021 May 5;18(9):4911. doi: 10.3390/ijerph18094911 (PMC8125545; doi:10.3390/ijerph18094911)
Supplement: Supplementary file 1 [file ijerph-18-04911-s001.zip › ijerph-1158351-supplementary.pdf]

Supplemental Figure 1. PRISMA flow diagram

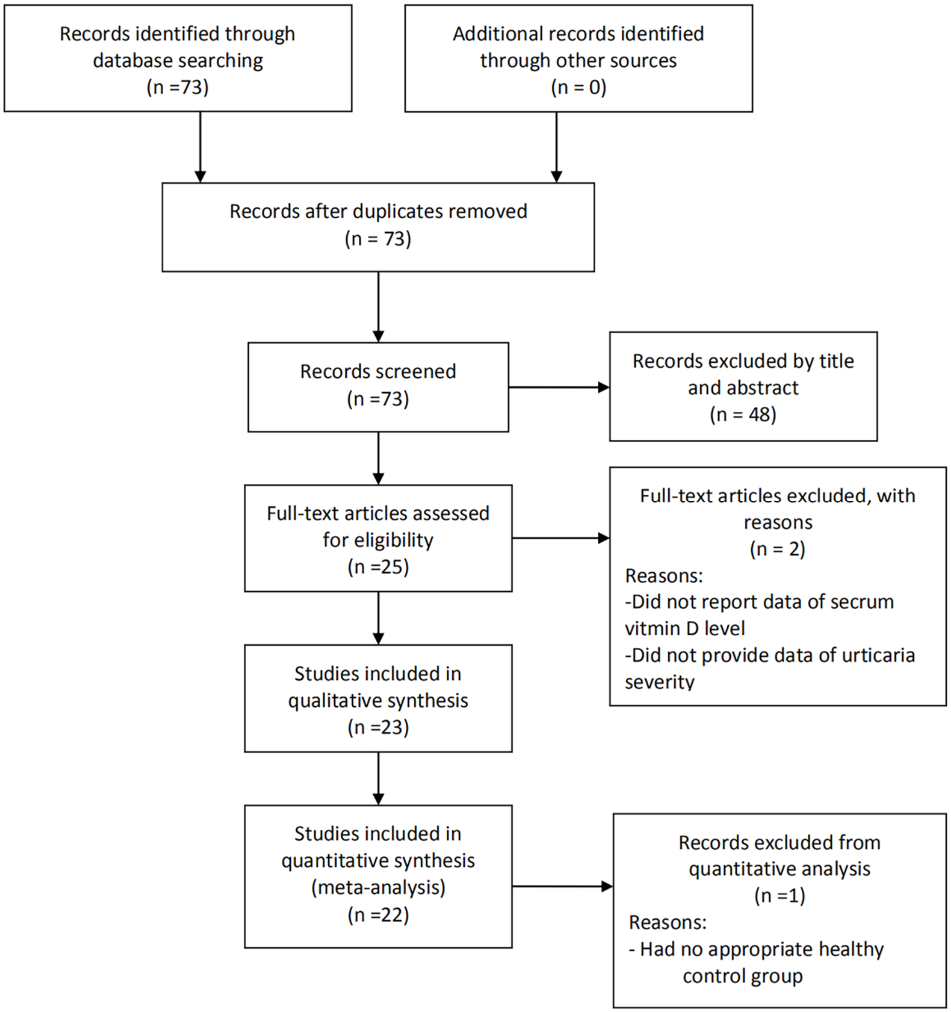

Supplemental Figure 2. Forest Plot for sub-analysis of serum vitamin D level comparison.

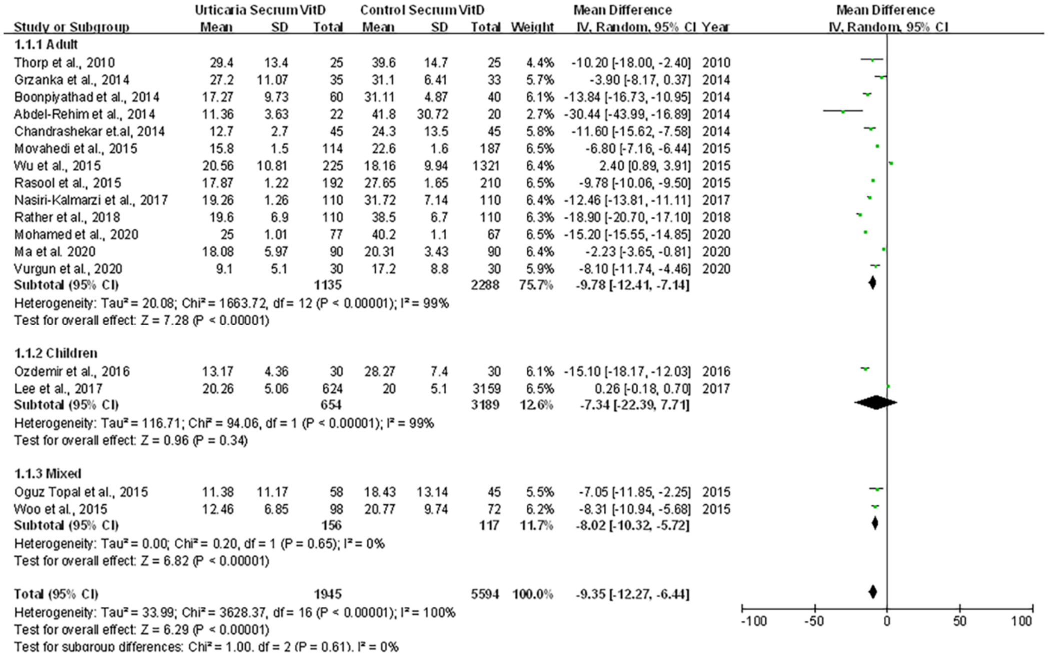

(a)

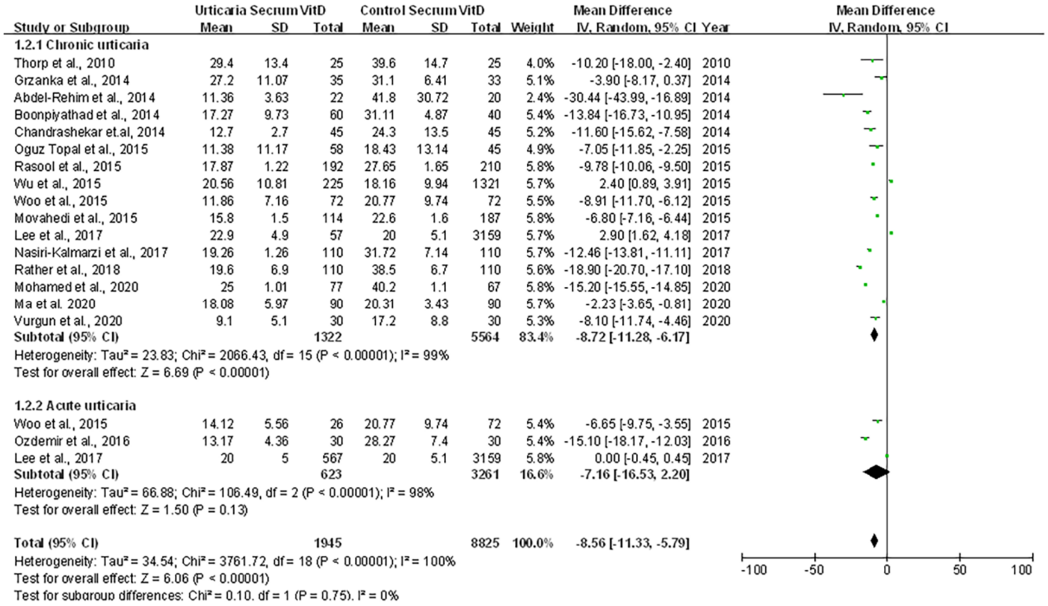

(b)

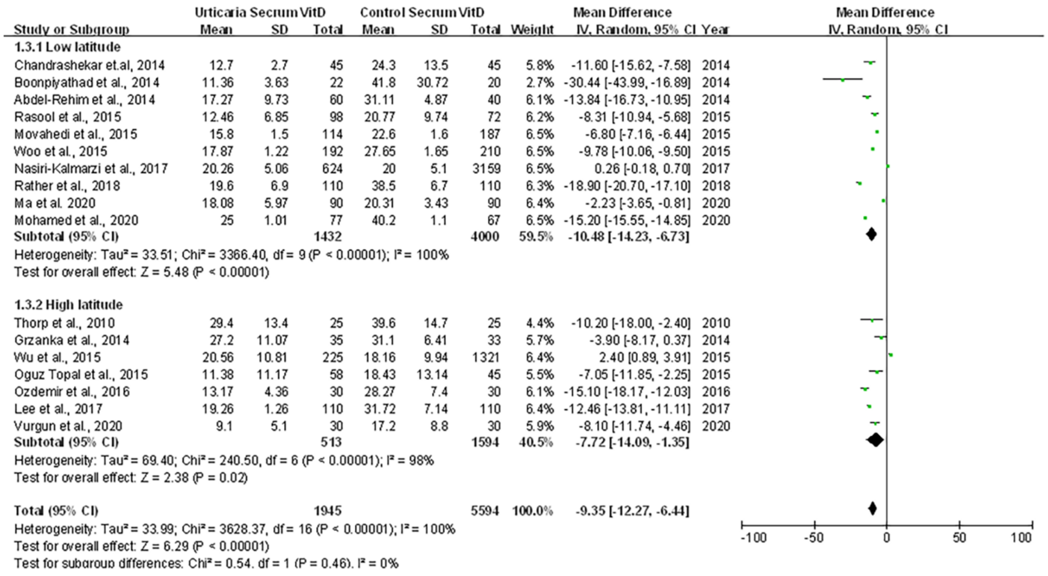

(c)

Fig. 2 (a) Subgrouped by age (b) Subgrouped by disease type (c)Subgrouped by latitude

Supplemental Figure 3. Forest Plot for sub-analysis of vitamin D deficiency and urticaria.

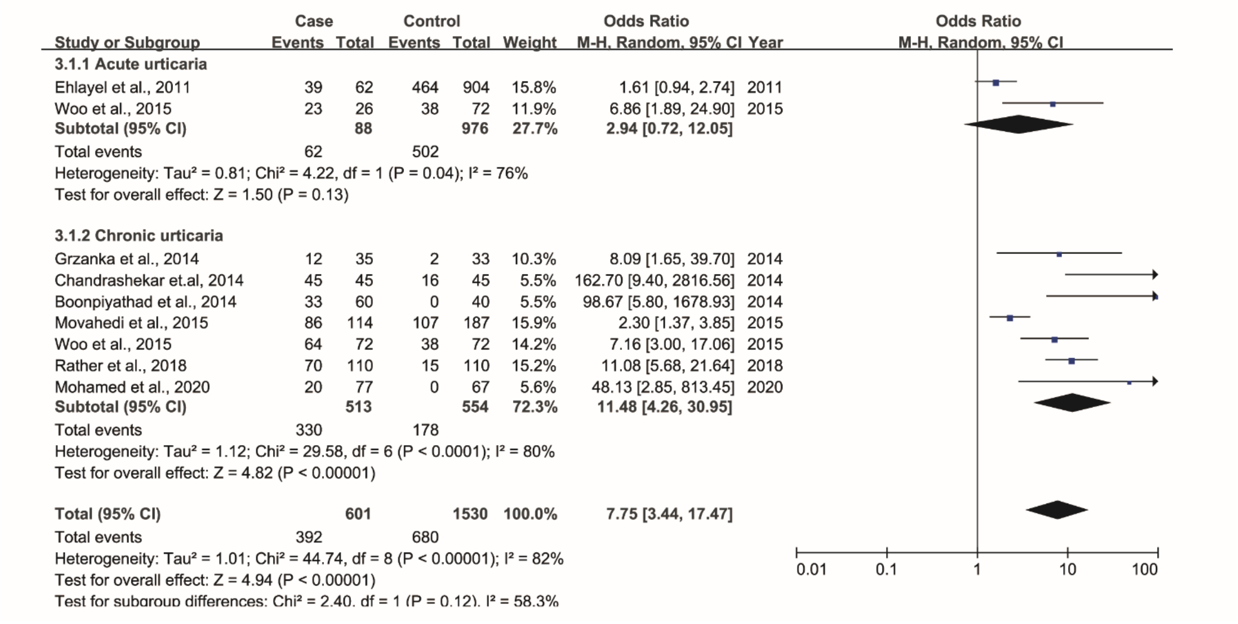

(a)

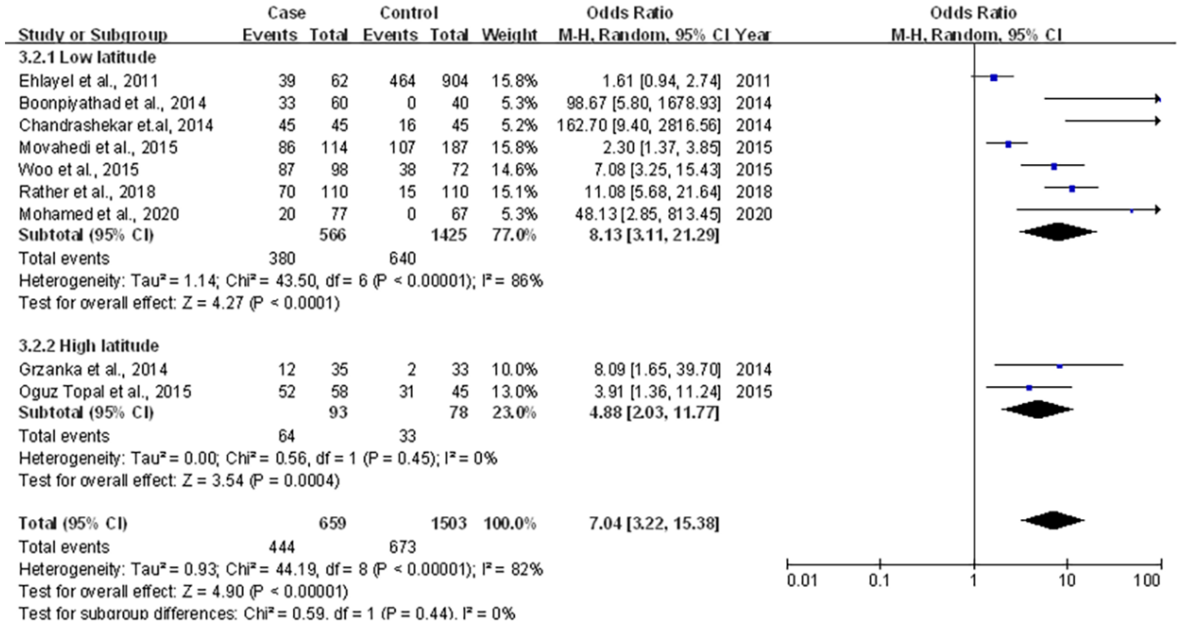

(b)

Fig. 3 (a) Subgrouped by disease type (b) Subgrouped by latitude

Supplemental Figure 4. Forest Plot for changes in clinical urticarial assessments after vitamin D supplementation.

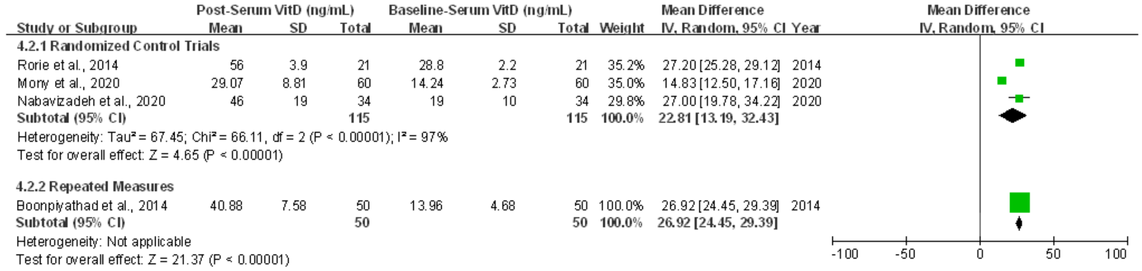

(a)

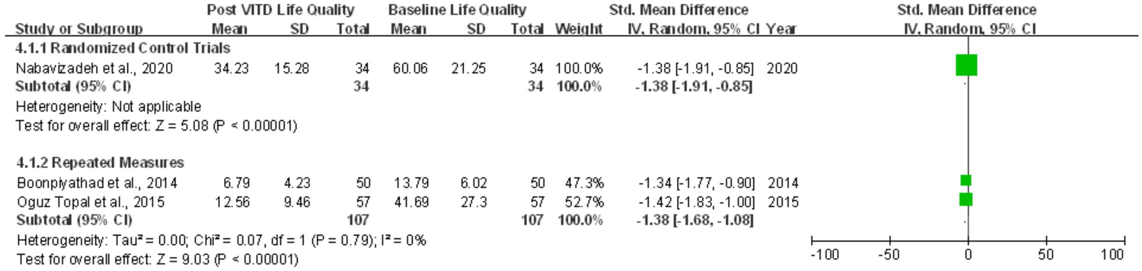

(b)

Fig. 4 (a) changes in serum 25(OH)VitD levels (b) changes in life quality scores

Supplemental Figure 5. Risk of bias

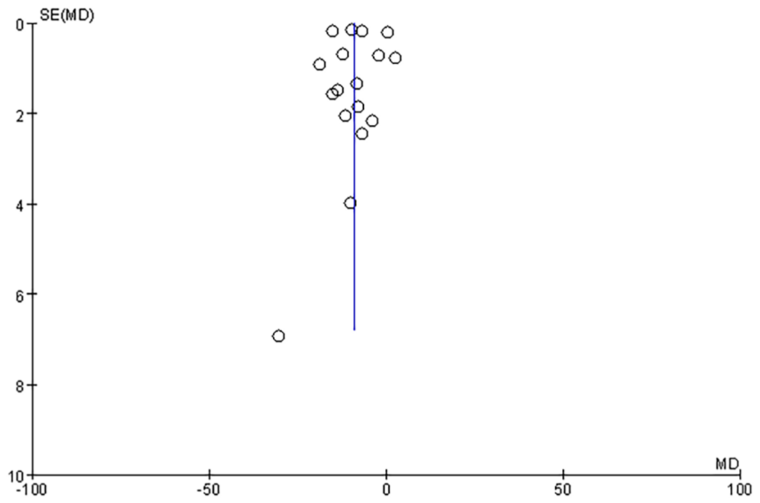

(a)

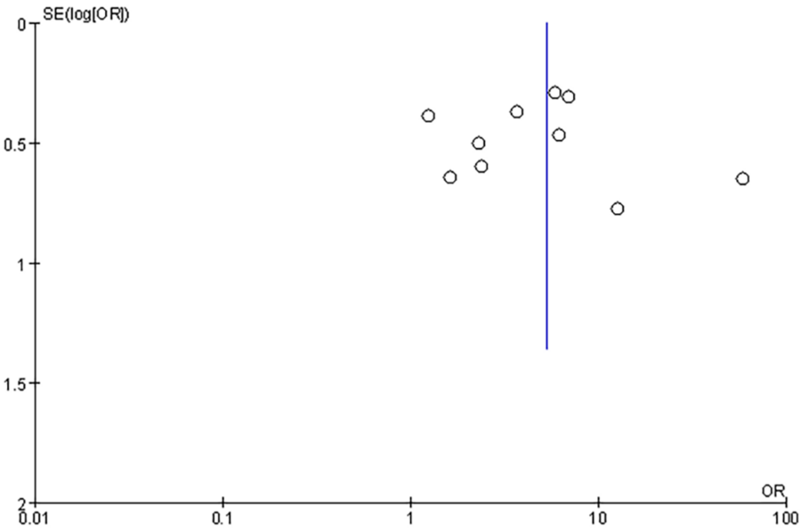

(b)

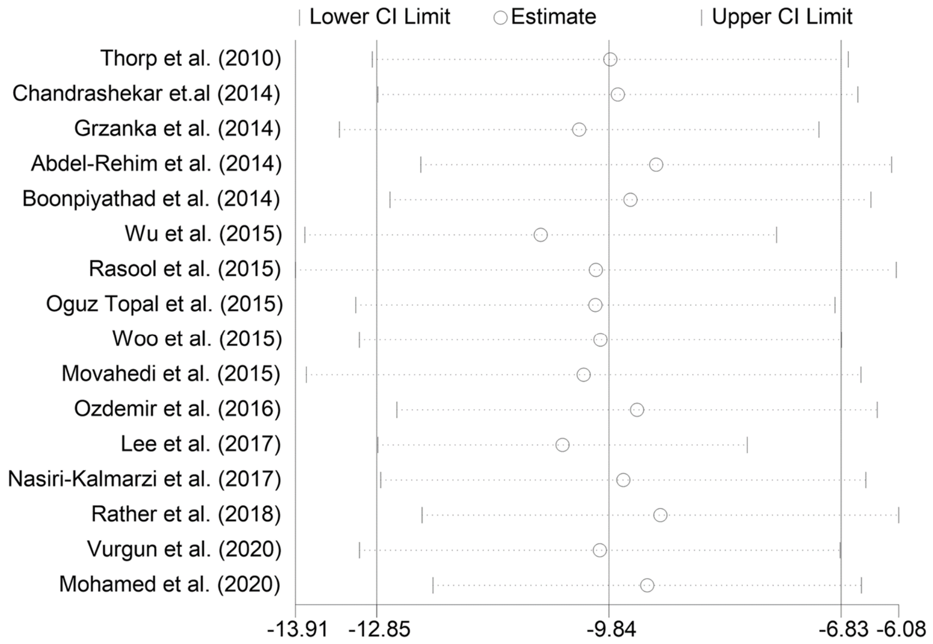
Lower CI Limit
Estimate
Upper CI Limit
Thorp et al. (2010)
Chandrashekar et.al (2014)
Grzanka et al. (2014)
Abdel-Rehim et al. (2014)
Boonpiyathad et al. (2014)
Wu et al. (2015)
Rasool et al. (2015)
Oguz Topal et al. (2015)
Woo et al. (2015)
Movahedi et al. (2015)
Ozdemir et al. (2016)
Lee et al. (2017)
Nasiri-Kalmarzi et al. (2017)
Rather et al. (2018)
Vurgun et al. (2020)
Mohamed et al. (2020)
-13.91
-12.85
-9.84
-6.83
-6.08

(c)

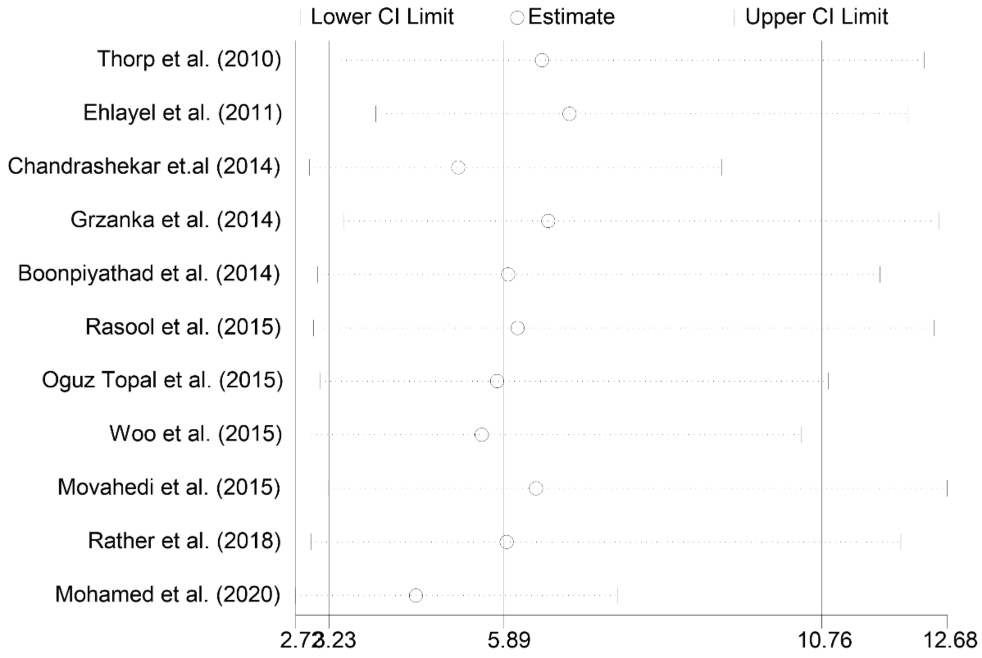
Lower CI Limit
Estimate
Upper CI Limit
Thorp et al. (2010)
Ehlayel et al. (2011)
Chandrashekar et.al (2014)
Grzanka et al. (2014)
Boonpiyathad et al. (2014)
Rasool et al. (2015)
Oguz Topal et al. (2015)
Woo et al. (2015)
Movahedi et al. (2015)
Rather et al. (2018)
Mohamed et al. (2020)
2.73
3.23
5.89
10.76
12.68

(d)

Supplemental Figure 6. The lack of association of serum vitamin D levels with acute urticaria and children urticaira.

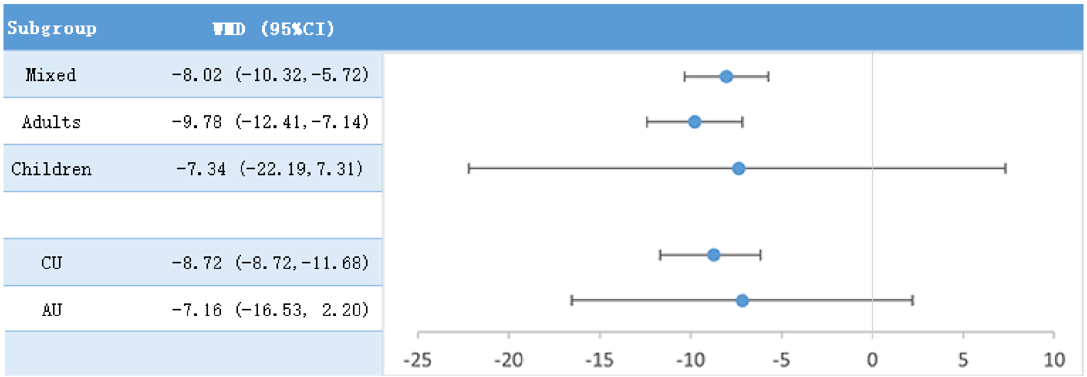

CU: chronic urticaria; AU: acute urticaria

Supplemental Table1. The methodological quality of studies included in the final analysis.

(a) Quality of the case-control studies based on the Newcastle–Ottawa Scale.

Study	Selection (Score)				Comparability (Score)	Exposure (Score)			Total Score
	Adequate definition of patient cases	Representativeness of the cases	Selection of Controls	Definition of Controls	Control for important factor or additional factor	Ascertainment of exposure (blinding)	Same method of ascertainment for participants	Non-response rate	
Thorp et al.[1], 2010	1	1	0	0	0	1	0	1	4
Ehlayel et al. [2], 2011	1	0	1	1	1	1	1	1	7
Chandrashekar et al.[3], 2014	1	1	0	1	1	1	1	1	6
Grzanka et al.[4], 2014	1	1	0	0	1	1	1	1	6
Abdel-Rehim et al.[5], 2014	1	1	0	0	1	1	1	1	6
Boonpiyathad et al.[6], 2014	1	1	0	0	1	1	1	1	6
Wu et al.[7], 2015	1	1	1	0	0	1	1	0	5
Rasool et al.[8], 2015	1	1	0	1	1	1	1	1	7

Oguz Topal et al.[9], 2015	1	1	0	0	1	1	1	1	6
Woo et al.[10], 2015	1	1	1	0	1	1	1	1	7
Movahedi et al.[11], 2015	1	1	0	1	1	1	1	1	7
Ozdemir et al.[12], 2016	1	1	0	1	1	1	1	1	7
Lee et al.[13], 2017	1	1	0	1	1	1	1	1	7
Nasiri-Kalmarzi et al.[14], 2017	1	1	0	1	1	1	1	1	7
Rather et al.[15], 2018	1	1	0	1	1	1	1	1	7
Vurgun et al.[16], 2020	0	1	0	1	2	1	1	0	6
Mohamed et al.[17], 2020	1	1	1	1	0	1	1	0	6
Ma et al.[18], 2020	1	1	1	1	2	1	1	0	8

(b) Quality and evidence level of the clinical trials based on the Jadad scale.

**Study**	**1.Sequence generation**	**2.Allocation concealment**	**3.Blinding**	**4.Incomplete outcome data**	**5.Selective outcome reporting**
Rorie et al., 2014[19]	Low risk	Low risk	Low risk	Low risk	Unclear
Ariaee et al. [20]	High risk	Unknown risk	High risk	Low risk	Low risk
Mony et al., 2020[21]	Low risk	Low risk	Low risk	Low risk	Low risk
Nabavizadeh et al., 2020[22]	Low risk	Low risk	Low risk	Low risk	Unclear

References:

[1]. Thorp WA, Goldner W, Meza J, Poole JA. Reduced vitamin D levels in adult subjects with chronic urticaria[J]. J Allergy Clin Immunol. 2010,126(2):413; author reply -4.

[2]. Ehlayel MS, Bener A, Sabbah A. Is high prevalence of vitamin D deficiency evidence for asthma and allergy risks?[J]. Eur Ann Allergy Clin Immunol. 2011,43(3):81-8.

[3]. Chandrashekar L, Rajappa M, Munisamy M, Ananthanarayanan PH, Thappa DM, Arumugam B. 25-Hydroxy vitamin D levels in chronic urticaria and its correlation with disease severity from a tertiary care centre in South India[J]. Clin Chem Lab Med. 2014,52(6):e115-8.

[4]. Grzanka A, Machura E, Mazur B, Misiolek M, Jochem J, Kasperski J, et al. Relationship between vitamin D status and the inflammatory state in patients with chronic spontaneous urticaria[J]. J Inflamm (Lond). 2014,11(1):2.

[5]. Abdel-Rehim AS, Sheha DS, Mohamed NA. Vitamin D level among Egyptian patients with chronic spontaneous urticaria and its relation to severity of the disease[J]. Egypt J Immunol. 2014,21(2):85-90.

[6]. Boonpiyathad T, Pradubpongsa P, Sangasapaviriya A. Vitamin D supplements improve urticaria symptoms and quality of life in chronic spontaneous urticaria patients: A prospective case-control study[J]. Dermatoendocrinol. 2016,8(1):e983685.

[7]. Wu CH, Eren E, Ardern-Jones MR, Venter C. Association between Micronutrient Levels and Chronic Spontaneous Urticaria[J]. Biomed Res Int. 2015,2015:926167.

[8]. Rasool R, Masoodi KZ, Shera IA, Yosuf Q, Bhat IA, Qasim I, et al. Chronic urticaria merits serum vitamin D evaluation and supplementation; a randomized case control study[J]. World Allergy Organ J. 2015,8(1):15.

[9]. Oguz Topal I, Kocaturk E, Gungor S, Durmuscan M, Sucu V, Yıldırmak S. Does replacement of vitamin D reduce the symptom scores and improve quality of life in patients with chronic urticaria?[J]. J Dermatolog Treat. 2016,27(2):163-6.

[10]. Woo YR, Jung KE, Koo DW, Lee JS. Vitamin D as a Marker for Disease Severity in Chronic Urticaria and Its Possible Role in Pathogenesis[J]. Ann Dermatol. 2015,27(4):423-30.
[11]. Movahedi M, Tavakol M, Hirbod-Mobarakeh A, Gharagozlou M, Aghamohammadi A, Tavakol Z, et al. Vitamin D deficiency in chronic idiopathic urticaria[J]. Iran J Allergy Asthma Immunol. 2015,14(2):222-7.
[12]. Ozdemir B, Köksal BT, Karakaş NM, Ozbek OY. Serum vitamin D levels decrease in children with acute urticaria[J]. Allergol Immunopathol (Madr). 2016,44(6):512-6.
[13]. Lee SJ, Ha EK, Jee HM, Lee KS, Lee SW, Kim MA, et al. Prevalence and Risk Factors of Urticaria With a Focus on Chronic Urticaria in Children[J]. Allergy Asthma Immunol Res. 2017,9(3):212-9.
[14]. Nasiri-Kalmarzi R, Abdi M, Hosseini J, Babaei E, Mokarizadeh A, Vahabzadeh Z. Evaluation of 1,25-dihydroxyvitamin D3 pathway in patients with chronic urticaria[J]. Qjm. 2018,111(3):161-9.
[15]. Rather S, Keen A, Sajad P. Serum Levels of 25-hydroxyvitamin D in Chronic Urticaria and its Association with Disease Activity: A Case Control Study[J]. Indian Dermatol Online J. 2018,9(3):170-4.
[16]. Vurgun E, Memet B, Kocaturk E, Guntas G. 25-hydroxyvitamin D levels are low but not associated with disease activity in chronic spontaneous urticaria and depression[J]. Bratisl Lek Listy. 2020,121(9):675-9.
[17]. Mohamed AA, Hussein MS, Salah EM, Eldemery A, Darwish MM, Ghaith DM, et al. Efficacy and safety of active vitamin D supplementation in chronic spontaneous urticaria patients[J]. J Dermatolog Treat. 2020:1-6.
[18]. Ma Y, Xiang Z, Yao X, Li C, Wu J, Feng S, et al. Associations between vitamin D receptor gene polymorphisms and chronic spontaneous urticaria in Chinese Han population[J]. Postepy Dermatol Alergol. 2020,37(2):250-4.
[19]. Rorie A, Goldner WS, Lyden E, Poole JA. Beneficial role for supplemental vitamin D3 treatment in chronic urticaria: a randomized study[J]. Ann Allergy Asthma Immunol. 2014,112(4):376-82.
[20]. Ariaee N, Zarei S, Mohamadi M, Jabbari F. Amelioration of patients with chronic spontaneous urticaria in treatment with vitamin D supplement[J]. Clin Mol Allergy. 2017,15:22.
[21]. Mony A, Chandrashekar L, Rajappa M, Munisamy M, Sahoo JP, Selvarajan S. Effect of vitamin D supplementation on clinical outcome and biochemical profile in South Indian population with vitamin D-deficient chronic urticarial - A randomized double-blind placebo controlled trial[J]. Clin Chim Acta. 2020,504:1-6.
[22]. Nabavizadeh SH, Alyasin S, Esmaeilzadeh H, Mosavat F, Ebrahimi N. The effect of vitamin D add-on therapy on the improvement of quality of life and clinical symptoms of patients with chronic spontaneous urticaria[J]. Asian Pac J Allergy Immunol. 2020.
